# Genome analysis of the thermoacidophilic archaeon *Acidianus copahuensis* focusing on the metabolisms associated to biomining activities

**DOI:** 10.1186/s12864-017-3828-x

**Published:** 2017-06-06

**Authors:** María Sofía Urbieta, Nicolás Rascovan, Martín P. Vázquez, Edgardo Donati

**Affiliations:** 1 CINDEFI (CCT La Plata-CONICET, UNLP), Facultad de Ciencias Exactas, Universidad Nacional de La Plata, Calle 47 y 115, 1900 La Plata, Argentina; 2 Instituto de Agrobiotecnología de Rosario (INDEAR), CONICET, Predio CCT, Rosario, Argentina; 3 Calle 50, entre 115 y 116, N° 227, La Plata, Buenos Aires, Argentina

**Keywords:** *Acidianus copahuensis*, Thermoacidophilic archaea, Biomining genes

## Abstract

**Background:**

Several archaeal species from the order Sulfolobales are interesting from the biotechnological point of view due to their biomining capacities. Within this group, the genus *Acidianus* contains four biomining species (from ten known *Acidianus* species), but none of these have their genome sequenced. To get insights into the genetic potential and metabolic pathways involved in the biomining activity of this group, we sequenced the genome of *Acidianus copahuensis* ALE1 strain, a novel thermoacidophilic crenarchaeon (optimum growth: 75 °C, pH 3) isolated from the volcanic geothermal area of Copahue at Neuquén province in Argentina. Previous experimental characterization of *A. copahuensis* revealed a high biomining potential, exhibited as high oxidation activity of sulfur and sulfur compounds, ferrous iron and sulfide minerals (e.g.: pyrite). This strain is also autotrophic and tolerant to heavy metals, thus, it can grow under adverse conditions for most forms of life with a low nutrient demand, conditions that are commonly found in mining environments.

**Results:**

In this work we analyzed the genome of *Acidianus copahuensis* and describe the genetic pathways involved in biomining processes. We identified the enzymes that are most likely involved in growth on sulfur and ferrous iron oxidation as well as those involved in autotrophic carbon fixation. We also found that *A. copahuensis* genome gathers different features that are only present in particular lineages or species from the order Sulfolobales, some of which are involved in biomining. We found that although most of its genes (81%) were found in at least one other Sulfolobales species, it is not specifically closer to any particular species (60–70% of proteins shared with each of them). Although almost one fifth of *A. copahuensis* proteins are not found in any other Sulfolobales species, most of them corresponded to hypothetical proteins from uncharacterized metabolisms.

**Conclusion:**

In this work we identified the genes responsible for the biomining metabolisms that we have previously observed experimentally. We provide a landscape of the metabolic potentials of this strain in the context of Sulfolobales and propose various pathways and cellular processes not yet fully understood that can use *A. copahuensis* as an experimental model to further understand the fascinating biology of thermoacidophilic biomining archaea.

**Electronic supplementary material:**

The online version of this article (doi:10.1186/s12864-017-3828-x) contains supplementary material, which is available to authorized users.

## Background

Biomining comprises technological processes (bioleaching and biooxidation) that use microorganisms, usually bacteria and archaea, to enhance the recovery of metals from insoluble ores mostly composed of metal sulfides. For the solubilization of sulfides, two conditions are required: an oxidizing agent and an acidic medium to maintain the removed metal cations in solution. Both conditions can be met by acidophilic iron- and sulfur oxidizing microorganisms; they can oxidize ferrous iron to ferric iron (a powerful oxidizing agent), and also oxidize metal sulfides and sulfur compounds to sulfuric acid [1]. Most of the commercial applications are implemented at moderate temperatures, below 50 °C, mainly because the firsts and best characterized bioleaching species are mesophiles. However, a higher operational temperature would be significantly beneficial as it would allow a reduction in the energy used for cooling the system (sulfur oxidation reactions are exothermic, causing a serious increase in temperature in bioreactors and inside the heaps) and would decrease the inconveniencies associated to mineral surface passivation [2]. Probably the most relevant example is the case of chalcopyrite (CuFeS_2_), a mineral species that accounts for approximately 70% of the world’s copper reserves [3] and is highly recalcitrant to chemical or mesophilic biological leaching [4]. In the past few years, several studies have shown that thermoacidophilic archaea are able to obtain faster solubilization rates and higher copper recovery yields than most used mesophilic bioleaching bacteria [5–7].

The physiological, biochemical and genetic characterization of thermoacidophilic archaea, especially the features related to biomining, became a topic of interest some years ago and some advances were made on elucidating the genes and metabolic pathways involved in the oxidation of sulfur compounds and ferrous iron. However, none of them are yet completely understood. The key enzymes for sulfur oxidation in thermoacidophilic archaea, the sulfur oxygenase reductase (SOR) and the thiosulfate quinone oxidoreductase (TQO), have been characterized in *Acidianus ambivalens* [8]. Regarding iron oxidation, a cluster of genes up-regulated when cultures were grown in ferrous iron was identified in *Sulfolobus metallicus*; thus this cluster, named *fox*, was directly linked to ferrous iron metabolism [9]. These genes are not present in other *Sulfolobus* species that do not oxidize iron. Some other biomining related features were also identified in the genome of *Metallosphaera sedula,* such as carbon fixation, metal resistance, and adhesion mechanisms [10]. Despite the light that these works shed into the unexplored bioleaching mechanisms of thermophilic archaea, many aspects of their metabolisms remain still unclear. The analysis of new genomes from this group, together with further experimental characterization will undoubtedly bring new insights into the biology of these organisms.

*A. copahuensis* is a novel thermoacidophilic archaeon from the domain Crenarchaeota and the order Sulfolobales*,* isolated by our group from the acidic Copahue geothermal area in the Northwest corner of the Cordillera de los Andes in Neuquén province (Argentina). It has shown a great physiological flexibility by growing in a temperature range of 55 °C to 80 °C and pH range from 1 to 5, with optimum conditions at 75 °C and pH 3, respectively [11]. Its metabolic features make it an excellent candidate for biomining of sulfide minerals as it is able to oxidize diverse sulfur compounds (sulfur, tetrathionate and metal sulfides such as pyrite and chalcopyrite), and ferrous irons, either heterotrophically or autotrophically, being the latter a valuable attribute in mining environments, where organic carbon is often limited. We have experimentally shown that *A. copahuensis* is able to recover a 100% of copper in the bioleaching of a chalcopyrite concentrate [7]. In addition, *A. copahuensis* can grow in anaerobic conditions using sulfur or hydrogen as electron donors and ferric iron or sulfur as electron acceptors, an essential adaptation for the anoxic conditions found below heaps surface [12]. In the present work we characterized the genome of this remarkable biomining candidate and the genes associated to its capabilities, such as the oxidation and reduction of sulfur and iron compounds, electron transport, carbon fixation, tolerance and resistance to heavy metals and metalloids. We also performed a comprehensive comparison of *A. copahuensis* genome with all other available genomes from the order Sulfolobales and found that it groups different features that are only found within specific genera of this order.

## Results and discussion

### *Acidianus copahuensis* within the order Sulfolobales

A total of 2559 genes were predicted in *Acidianus copahuensis* ALE1 strain (DSM 29038) genome using the RAST annotation server. The comparison to all other available genomes of the order Sulfolobales at the whole genome level using an in silico DDH method showed only a 30% similarity to the closest genome and only 15% to *Acidianus hospitalis*, the other sequenced species within the genus *Acidianus* (Table 1).

**Table 1:** Digital DDH estimation in silico of *Acidianus copahuensis* genome against all other available Sulfolobales genomes

Query genome	Reference genome	DDH (%)	+/−	Distance	Prob. DDH > = 70%	G + C difference
*Acidianus copahuensis*	*Sulfolobales Acd1*	30.1	2.45	0.1416	0.11	16.04
*Acidianus copahuensis*	*Sulfolobus tokodaii*	28.2	2.43	0.1526	0.05	2.85
*Acidianus copahuensis*	*Sulfolobus solfataricus*	25.3	2.4	0.1719	0.01	0.15
*Acidianus copahuensis*	*Sulfolobus acidocaldarius*	23.8	2.38	0.1833	0	1.07
*Acidianus copahuensis*	*Sulfolobus islandicus*	21.9	2.35	0.2007	0	0.53
*Acidianus copahuensis*	*Metallosphaera sedula*	20.6	2.32	0.2133	0	10.59
*Acidianus copahuensis*	*Metallosphaera hakonensis*	19.4	2.29	0.2263	0	7.68
*Acidianus copahuensis*	*Sulfolobus metallicus*	19.2	2.28	0.2294	0	2.97
*Acidianus copahuensis*	*Metallosphaera cuprina*	18.6	2.27	0.2368	0	6.36
*Acidianus copahuensis*	*Metallosphaera yellowstonensis*	17.9	2.24	0.2453	0	12.11
*Acidianus copahuensis*	*Acidianus hospitalis*	15.7	2.16	0.278	0	1.49
*Acidianus copahuensis*	*Sulfolobales AZ1*	15.6	2.15	0.2804	0	11.35

**Fig. 1:**
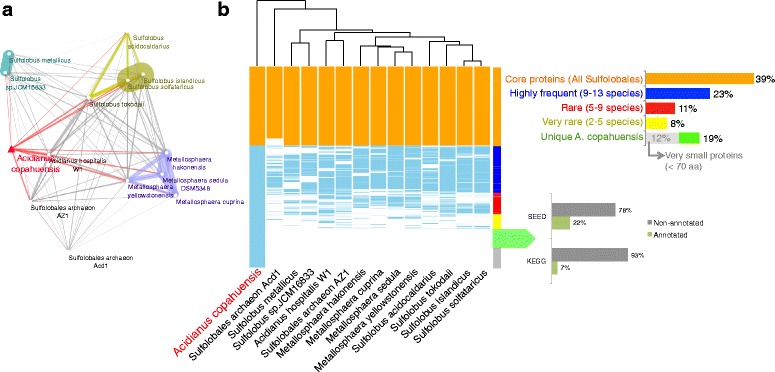
*Acidianus copahuensis* proteins compared to all other Sulfolobales species. **a** Network analysis representing the results of an “all vs. all” BLASTP comparison of all Sulfolobales proteins from sequenced genomes. Only hits with more than 65% of both proteins aligned and E-value lower that 1E-20 were considered as a match between two proteins. The line width is proportional to the number of proteins shared by two species. **b** Heatmap analysis representing the *Acidianus copahuensis* proteins that were found in other Sulfolobales species based on the BLASTP comparison mentioned above. Proteins were classified depending on the number of genomes where each protein is present (see heatmap side bar and the upper-right bar chart as a reference for the categories used). Clustering was performed using the Euclidean distance

According to a network analysis comparing all proteins from Sulfolobales genomes, *Acidianus copahuensis* is not closer to any particular genus among Sulfolobales (Fig. 1a). It shares around two thirds (min: 50%, max: 68%, avg.: 64%) of its proteins with each of the other Sulfolobales species (Additional file 1: Figure S1) and 39% of them (1003) are core proteins present in all genomes (Fig. 1b, Additional file 2: Table S1). In fact, the proportion of core proteins would be even higher (62%) if we also consider those proteins that were found in most (≥ 9 of 14) Sulfolobales species (Fig. 1b, Additional file 2: Table S1).

On the other hand, 19% (469) of *A. copahuensis* proteins were not found in any other available genome, and therefore correspond to unique features of this species (Fig. 1b, Additional file 2: Table S1). However, most of these proteins could not be annotated by either SEED or KEGG systems (Fig. 1b). Among the few that were annotated, we could not find key enzymes of known metabolisms, and therefore further experimental work will be required to determine their metabolic roles. Nevertheless, considering that these genes were likely selected specifically in *A. copahuensis*, we could hypothesize that they provide advantages to survive under the extreme and particular environmental conditions of Copahue geothermal system where this species naturally grows. Finally, we found that another 19% of *A. copahuensis* proteins were rarely found in Sulfolobales genomes (only in 2 to 9 of 14 genomes) (Figure 1b, Additional file 1: Figure S1). Some of these proteins are involved in bioleaching activities and are further discussed in the following sections.

All together, these results indicate that *A. copahuensis* is a novel species distantly related to *A. hospitalis* and with multiple features that are only found in particular genomes of the order Sulfolobales.

#### Sulfur compounds metabolism

The dissimilatory oxidation of elemental sulfur and sulfur compounds is one of the most important metabolic processes in acidophilic microorganisms that grow in volcanic environments. From the biotechnological point of view, medium acidification as a consequence of biooxidation of sulfur compounds is one of the main reasons for using acidophiles in biomining. As mentioned before, *A. copahuensis* is a sulfur oxidizing archaeon able to grow using sulfur, tetrathionate or sulfide minerals as energy sources.

The sulfur oxygenase reductase (SOR) has been considered the key enzyme in the thermoacidophilic archaeal sulfur oxidation pathway [13]; it simultaneously oxidizes and reduces sulfur (disproportionation) coupled to an oxygenase reaction in aerobic conditions at high temperature according to Eq. 1.

1 $$ 5\ {\mathrm{S}}^{{}^{\circ}} + {\mathrm{O}}_2 + {4\ \mathrm{H}}_2\mathrm{O}<=>{{\ \mathrm{S}\mathrm{O}}_3}^{-2} + {\mathrm{S}}_2{{\mathrm{O}}_3}^{-2} + {2\ \mathrm{H}\mathrm{S}}^{-} + {6\ \mathrm{H}}^{+} $$

**Fig. 2:**
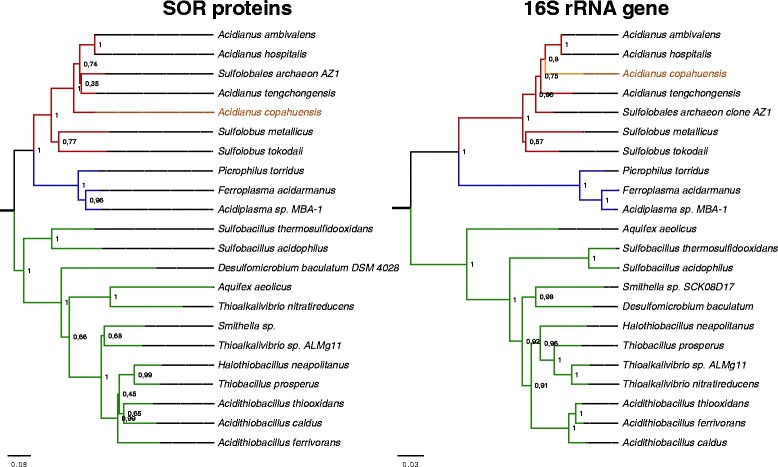
Phylogeny of Sulfolobales based on SOR proteins and 16S rRNA genes. Phylogenetic trees were obtained by the Maximum Likelihood method for all known SOR proteins found in NCBI database (**a**) and the corresponding 16S rRNA genes (**b**) in the same organisms. Bootstrap supports for nodes were obtained using 1000 repetitions and are expressed as the proportion of times (in decimals) that each node was supported. Archaea branches from the phylum Euryarchaeota are colored in blue and from the phylum Crenarchaeota in red while Bacteria branches are in green

In the genome of *A. copahuensis* we detected a SOR homologous gene that encodes a 308 amino acid protein with 85% similarity to the SOR of *A. ambivalens* [14] and 88% with that found in *A. tengchongensis* [15]. We searched for homologous SOR sequences in the available genomes of NCBI database and we found a total of 21 other SOR-like sequences. These proteins are encoded by species from distant taxonomic groups such as some from order Sulfolobales within the phylum Crenarchaeota, order Thermoplasmatales within the phylum Euryarchaeota and the phyla Firmicutes, Aquificales, and Proteobacteria (Beta, Delta, and Gamma) within the domain Bacteria. Only 4 from the 14 available Sulfolobales genomes (and two other Sulfolobales species with no genome sequenced) contain SOR proteins, indicating sulfur oxidation through this enzyme may be limited to a reduced number of species within this order. However, other Sulfolobales species such as *M. sedula* are able to oxidize sulfur using other proteins than SOR [16]. A phylogenetic analysis of the SOR proteins showed a very similar topology to the corresponding tree of 16S rRNAs genes from the same species [17]. Since the 16S rRNA gene is a highly conserved, ancestral and essential gene, it is normally used to estimate the evolutionary origin of prokaryotic species. Genes that display similar topologies as those found on the 16S rRNA gene are likely to have evolved within their lineages and not acquired, for example, by horizontal gene transfer. Therefore the presence of SOR proteins in Bacteria, Euryarchaeota and Chrenarchaeota and the congruence between SOR and 16S rRNA genes phylogenies, indicates that SOR genes might have evolved from ancient prokaryotic lineages (Fig. 2). While the tree topologies of 16S rRNA genes and SOR proteins are almost identical within the Archaea domain, incongruences on different branches within the Bacterial lineage suggest that horizontal gene transfer events might have occurred more frequently in Bacteria. These results may indicate that sulfur oxidation through the SOR enzyme was a bioenergetic metabolism present in ancient forms of life, which was used to grow under the extreme environmental conditions of the primitive Earth. Since SOR-coding species are usually found in extreme environments with common conditions to those that were once present in primitive Earth, we can hypothesize that these genes played key metabolic role that was positively selected through time in the microbial lineages that live under these conditions.

*A. copahuensis* presents homologous genes for the enzymes that oxidize the SOR reaction products (SO_3_^−2^, S_2_O_3_^−2^, HS^−^), which allow coupling sulfur oxidation to electron transport and substrate level phosphorylation [18] (Additional file 3: Table S2). We found a putative sulfide:quinone oxidoreductase (SQR), a flavoprotein that oxidizes H_2_S and transfers the electrons to the respective quinone, that presents over 80% identical amino acid positions with SQR of *A. hospitalis*, *A. ambivalens* and *M. sedula*. Regarding SQR, it is worth mentioning that although multiple homologous genes have been found in archaeal genomes, so far there are no experimental confirmations of the SQR activity of these genes [13]. *A. copahuensis* genome contains a genomic locus coding for the two subunits of the thiosulfate:quinone oxidoreductase (TQO) (also known as *tqoAB* complex or *doxA/doxD* genes), which showed between 77 and 87% sequence similarity with TQO subunits from *A. ambivalens, A. hospitalis, S. tokodaii,* and *M. sedula.* To complete the oxidation pathway of the SOR reaction products, we searched in the *A. copahuensis* genome for putative sulfite:acceptor oxidoreductase (SAOR) enzymes using the reported sequence of the molybdopterin binding subunit A of the sulfite:cytochrome c oxidoreductase from *Starkeya novella* DSM 506 (acc number AAF64400, encoded by *sorA* gene) [19]. We identified a 203 amino acid homologous protein that also shows high sequence similarity (60–70%) to other oxidoreductases reported in the genomes of different Sulfolobales, including the sulfite-oxidase molybdopterin protein, product of the *som* gene reported in *M. yellowstonensis* [20]. Further analyses will be needed to find whether the *A. copahuensis* protein has sulfite oxidoreductase activity and if so, to elucidate its mechanism of electron transfer and subcellular location. On the other hand, neither *A. copahuensis* nor the other Sulfolobales species contain homologous genes of the *sorB* gene that encodes a c-type cytochrome in *S. novella*. Other unidentified cytochromes could be involved in the transfer of the sulfite oxidase reaction electrons in Sulfolobales species.

Regarding the indirect mechanism of SO_3_^−2^ oxidation, we used the amino acid sequences of adenylylsulfate reductase and adenylylsulfate:phosphate adenylyltransferase reported in *Thiobacillus denitrificans* [21] for a BLAST search, but we could not find reliable hits in the genomes of *A. copahuensis* nor in *A. hospitalis*, the only other *Acidianus* with sequenced genome.

In the case of tetrathionate metabolism, we found homologous genes to the three tetrathionate hydrolase (*tth*) genes present in the TTH complex of *A. ambivalens* (CBY66038), which is responsible of transforming S_4_O_6_^−2^ into S, S_2_O_3_^−2^, and SO_4_^−2^ at acidic pH [22]. The finding of homologous *tth* genes in the genome of *A. copahuensis* is consistent with the experimental observation of cultures growing in S_4_O_6_^−2^ as sole energy source and producing visible amounts of sulfur. The TTH is not expressed in sulfur-grown cells and these genes were so far only found in S_4_O_6_^−2^ oxidizing obligatory or facultative chemolithoautotrophs [23]. Considering that *A. copahuensis* is able to grow chemolithoautotrophically on sulfur and on tetrathionate and all the genes that code for the required proteins (SOR, SQR, TQO, TTH) were found in the draft genome, we propose a mechanism where the electrons that remain available in the products from the SOR reaction can be channeled to terminal oxidases through a series of intermediate oxidizing enzymes (see Additional file 4: Figure S2 for more detail).

The mechanisms used by sulfur oxidizing thermoacidophilic archaea to incorporate sulfur and sulfur compounds into the cytoplasm are yet unclear. In certain bacteria, such as the phototrophic sulfur oxidizing *Allochromatium vinosum*, the Dsr system is involved in sulfur oxidation pathway [24] as well as in sulfur transport into the cytoplasm (DsrC, DsrEFH, TusA, DsrAB and a rhodanese-like protein) [25–27]. *A. copahuensis*, as the other Sulfolobales, does not have homologous genes to *dsrC* or *dsrAB* but it harbors genes coding for two putative DsrE like proteins (active-site subunit of the DsrEFH complex and key part in sulfur traffic in *A. vinosum*) with high similarities with DsrE-like proteins reported in *M. cuprina* genome [28]. These DsrE-like proteins are encoded in a gene cluster that also contains a *tusA* homologous gene and the components of the heterodisulfide reductase complex (*hdr*) in the same order that in *M. cuprina* genome (Additional file 3: Table S2 and Additional file 5: Figure S3). We searched for homologous genes of this cluster in *A. copahuensis* and in all other available Sulfolobales genomes and we found that all of them followed an almost identical organization (dihydrolipoamide dehydrogenase -, *dsrE3A-, dsrE2B+, tusA+, hdrC1+, hdrB1+, hdrA*+, Conserved hypothetical protein +, *hdrC2*+ and *hdrB2*+).

Experimental evidence indicated that DsrE and TusA of *M. cuprina* have the ability to mobilize S_2_O_3_^−2^ from S_4_O_6_^−2^ and TusA is possibly implicated in dissimilatory tetrathionate oxidation [see ref. [28] for more detail]. Moreover, transcription of *dsrE*-, *tusA*- and *hdr*-like genes was up regulated in *M. sedula* when sulfur or tetrathionate were provided as electron donors [29]. Heterodisulfide reductase (Hdr) is a key enzyme in the energy metabolism of methanogenic archaea and sulfate reducing microorganisms that catalyzes the reversible reduction of heterodisulfide bonds associated to energy conservation by the extrusion of protons across the membrane creating a proton motive force [30, 31]. In sulfur oxidizing prokaryotes the Hdr-like complex is proposed to be associated to the production of sulfite from TusA-bound sulfur, replacing the Dsr system (see ref. [24] for more detail). The HdrABC-like complex is also present in *At. ferrooxidans* and it was found up regulated when cells were grown in sulfur [32]. Due to the natural proton gradient between intra and extracellular media in *At. ferrooxidans* and some acidophilic sulfur oxidizing Sulfolobales, among others, it has been proposed that the Hdr complex might work in reverse, oxidizing disulfide intermediates to sulfite and transporting the electrons to the membrane quinol pool [32]. The Hdr of *Aquifex aeolicus* has been purified and characterized as a complex composed of at least five subunits: HdrA, HdrB1, HdrB2, HdrC1 and HdrC2; and even though the functioning and organization in vivo are still unknown, it is proposed to be a membrane attached enzyme able to produce sulfite from sulfur associated to TusA [33].

**Fig. 3:**
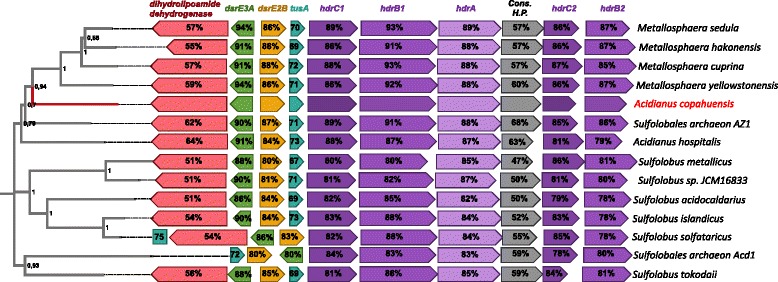
Organization of *dsrE*-*tusA*-*hdr*-like gene clusters in Sulfolobales. Genomic organization of the cluster is almost identical in all Sulfolobales to that shown by Liu et al. 2014 for the *M. cuprina* genome [28]. *Acidianus copahuensis* proteins were used as references to calculate the percentage similarity to corresponding proteins from all other species. The homologous genes from different species are represented using the same color. Arrow orientation indicates the orientation of the ORF in the genome and lengths are proportional to the real length of the protein. “Cons. H.P.” is an acronym for conserved hypothetical protein. Double bar indicates that the gene is located in a different genomic region. All proteins from each species were concatenated and the resulting poly-proteins were used to build a Maximum Likelihood phylogenetic tree, placed on the left of the figure. Bootstrap supports for nodes were obtained using 1000 repetitions and are expressed as the proportion of times (in decimals) that each node was supported

A phylogenetic analysis performed with the concatenated proteins of the *dsrE-tusA-hdr* cluster showed a topology consistent with the evolutionary history of Sulfolobales species (Fig. 3). This result indicates that this cluster was likely not spread by a recent horizontal gene transfer event. Moreover, considering the high protein similarities between species, it would also suggest that mutation rate is limited by a high selective pressure to maintain enzymatic functions. The genomic organization in *A. copahuensis* reinforces the idea that the close link between *tusA*, *hdr*, *dsrE*– like genes and lipoamide dehydrogenase encoding gene might indicate a functional association, including the transport of electrons coming from sulfane sulfur oxidation to NAD^+^ [28, 33]. A brief schematic representation of the potential sulfur compounds oxidizing activity of Hdr, TusA and DsrE is shown in Additional file 4: Figure S2. Further work will be needed to better understand the sulfur metabolic processes involving the *dsrE-tusA-hdr* cluster in Sulfolobales, for which *A. copahuensis* could serve as model.

Regarding sulfur anaerobic metabolism, we had experimentally observed that *A. copahuensis* is able to use H_2_ as electron donor to reduce sulfur into H_2_S [11]. The genome of *A. copahuensis* contains the 12-gene cluster corresponding to a membrane-bound periplasmic Ni/Fe hydrogenase generally associated to this mechanism in Crenarchaeota. These genes were also reported in *A. ambivalens* and are only partially present in four other Sulfolobales genomes (*M.sedula, M.yellowstonensis* and strains AZ1 and Acd1) [8, 34] (Table 2). According to a comprehensive bioinformatic study performed on this kind of enzymes, called Ips-hydrogenases, the putative structure of the enzyme (with still unknown function in vivo) would allow several electron transference reactions between H_2_ and sulfur compounds [35]. We did not find any hits to the *sreABCDE* cluster, coding for Sulfur reductases in *A. ambivalens* [8, 34]. This could indicate the presence of a different mechanism for sulfur reduction in *A. copahuensis* and opens the door to further study H_2_/S chemolithotrophic anaerobic metabolism.

**Table 2:** Comparison of the genes and proteins in the hydrogenase cluster described in *A. ambivalens* [72] with the correspondent homologous found in *A. copahuensis* genome

*Acidianus ambivalens*			Homologs in A. copahuensis				
Gene	Protein acc. Number (NCBI)	ORF (aa)	ORF (aa)	Protein acc. Number (NCBI)	Aa identical positions (%)	Prevalence in Sulfolobales (# of genomes)	Predicted function
*hynL*	CAC86887	628	633	EZQ01597	82	4 (M.S / M.Y / S.AZ1 / S.acd1)	membrane-bound NiFe hydrogenase large subunit
*hynS*	CAC86884	417	417	EZQ01596	85	4 (M.S / M.Y / S.AZ1 / S.acd1)	NiFe hydrogenase small FeS subunit
*isp1*	CAC86885	269	278	EZQ01616	68	2 (S.AZ1, S.acd1)	hydrogenase membrane anchor, heme b-binding
*isp2*	CAC86886	454	452	EZQ01617	74	2 (S.AZ1, S.acd1)	hypotetical FeS subunit
*hynZ*	CAC86889	201	206	EZQ01618	55	1 (S. acd1)	hypothetical protein from hydrogenase cluster
*hynY*	CAC86888	113	92	EZQ01598	70	1 (S. acd1)	Rieske-type FeS protein
*hypD*	CAC86890	394	395	EZQ01599	69	4 (M.S / M.Y / S.AZ1 / S.acd1)	hydrogenase maturation protein
*hypC*	CAC86891	101	104	EZQ01600	65	2 (S.AZ1, S.acd1)	hydrogenase maturation protein
*Hype*	CAC86892	339	335	EZQ01601	67	4 (M.S / M.Y / S.AZ1 / S.acd1)	hydrogenase maturation protein
*hypY*	CAC86893	70	72	EZQ01602	45	0	Unknown/hypothetical protein in hydrogenase cluster
*hypZ*	CAC86894	143	142	EZQ01603	64	2 (S.AZ1, S.acd1)	Unknown/hypothetical protein in hydrogenase cluster
*hoxM*	CAC86895	163	159	EZQ01604	54	2 (S.AZ1, S.acd1)	Maturation protease for HynL

The proteins are identified by their NCBI accession number. ORF (aa): indicates length of the open reading frame in amino acids. The prevalence in other Sulfolobales species was calculated based on the all vs. all BLASTP comparison used for Fig. 1. For some proteins, more than one hit was found in certain species (See Additional file 2: Table S1). Acronyms: M.S. (*Metallosphaera sedula*), M.Y (*Metallosphaera yellowstonensis*), S.AZ1 (*Sulfolobales archaeon AZ1*), S.acd1 (*Sulfolobales archaeon Acd1*). Aa: amino acids

#### Iron metabolism

Iron oxidation is common among acidophilic microorganisms, in both Bacteria and Archaea, and it is a required feature for biomining microorganisms. Their ability to continuously produce ferric iron, a powerful oxidizing agent, is essential to solubilize acid-insoluble metal sulfides like pyrite, molybdenite, and tungstenite. Due to their particular electron distribution and metal-sulfide bond configuration, these sulfides are resistant to proton attack. Only strong oxidizing agents like ferric iron are able to break the metal-sulfide bonds through a multistep mechanism called thiosulphate pathway (see [1] for more detail).

**Fig. 4:**
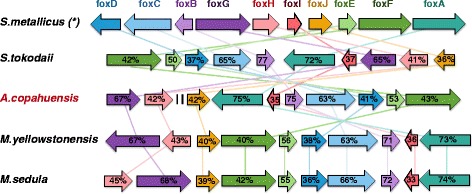
Organization of *fox* genes clusters in Sulfolobales. *Sulfolobus metallicus* genes were used as reference because it is the model organism where this complex was first described (*). Similarity of the amino acid sequences of all Sulfolobales with *fox* genes to the *S.metallicus* references was estimated using a BLASTP analysis. The homologous genes from different species are represented using the same color, arrow orientation indicates the orientation of the ORF in the genome and lengths are proportional to the real length of the proteins. Homologous genes are linked by lines to track changes in genome organization among species. Double bar indicates that the gene is located in a different genomic region

Autotrophic microbial iron oxidation is only well characterized in some bacteria, mainly in *At. ferrooxidans* [32, 36]. Although the mechanisms and enzymes involved in iron oxidation in thermoacidophilic archaea are not fully understood, significant advances have been made in the last years such as the identification of two membrane complexes in *Ferroplasma acidiphilum*; one that directly oxidizes ferrous iron and reduces molecular oxygen and a *ba* complex proposed to be involved in iron oxidation respiratory chains (uphill and downhill electron flow pathways) [37] or the proposal of the Mco multibluecopper oxidase protein of *M. yellostonensis* as an electron storage (like rusticyanin in *At. ferrooxidans*) and/or involved in electron transference between the Fox proteins [20]. The *foxABCDEFGHIJ* gene cluster was only detected in *Sulfolobus* and *Metallosphaera* species that can grow on ferrous iron or pyrite (the gene content and organizations differ between species). Furthermore, a high expression of some of them, particularly *foxA*, *B*, *C* and *D*, was observed in ferrous iron growth [9, 10, 20]. Briefly, the *fox* cluster codes for the two subunits of a putative heme copper oxidase (FoxA and B), a *b*-type cytochrome proposed as the direct oxidant of ferrous iron (Fox C and D), a Fe–S protein and other proteins of unknown function; many of these are proposed or confirmed membrane proteins [20, 38]. *A. copahuensis* is one of the few Sulfolobales species that can oxidize ferrous iron and pyrite and we have identified the ten *fox* genes, which most likely perform this function (Additional file 6: Table S3). Fig. 4 shows the particular organization of the *fox* cluster in *A. copahuensis* compared to the other iron oxidizing Sulfolobales. Since either all or none of the genes were found in Sulfolobales species, all genes are most likely essential for this metabolism.

*A. copahuensis* also bares homologous genes to the *cbsAB/soxNL* cluster (Additional file 3: Table S2), which codes for a cytochrome *ba* complex, analog to the *bc1* complex of bacteria and mitochondria [39], that is highly expressed in *M. sedula* grown on ferrous iron [10] and it is proposed to be involved in the uphill/downhill electron transport associated with chemolithotrophic iron oxidation [38]. *A. copahuensis* does not have homologous to the *mco* gene from *M. yellowstonensis*, however we identified a gene coding for a rusticyanin-like protein that is also found in other five species of Sulfolobales (Additional file 2: Table S1). Rusticyanin plays a key role in other iron oxidizing species as electron storage and branch point between uphill (NAD^+^ reduction) and downhill (O_2_ reduction) electron flow [40]. Rusticyanins are also similar to sulfocyanins (such as SoxE), which are also involved in electron transference, and for this reason they could be often confused with each other. The phylogenetic analysis of the rusticyanin from *A. copahuensis* showed a clear and well-supported clustering with known functional rusticyanin proteins (such as those found the bacteria *Acidithiobacillus ferrivorans*) and very distinct from sulfocyanins (including that from *A. copahuensis* itself) (Additional file 5: Figure S3). Most interestingly, it is found in Bacteria, Euryarchaeota and Crenarchaetoa, and the phylogenetic analysis supports an ancient origin of this protein. The fact that it is no longer widespread in the three lineages, suggests that it could have played essential roles in the metabolisms required to survive in the extreme conditions of ancient Earth, which are not longer present in modern days, but that are still required under the environmental conditions and the physiological needs of *A. copahuensis.*

The present findings make *A. copahuensis* a good candidate to study a divergent model of iron oxidation to that proposed in *M. yellowstonensis* [20] that involves the *fox* cluster and may use the rusticyanin-like gene instead of the *mco* gene to facilitate electron transport.

#### Oxidases and terminal oxidases

The oxidation of ferrous iron, sulfur, or sulfur compounds in aerobic organisms is necessarily linked to the respiratory chain and coupled to oxygen reduction and proton translocation. This coupling is executed by oxidases and terminal oxidases. Apart from the *fox* cluster already described, *A. copahuensis* genome presents homologous genes to most of the terminal oxidases so far described in *Sulfolobales* species (Additional file 3: Table S2). We found homologous of the *sox* genes in a cluster that codes for putative SoxA (quinol oxidases subunit II), SoxB (cytochromes *aa*_3_ subunit) and SoxC (quinol oxidase cytochrome *b* subunit) proteins, all with very high similarity to SoxABC proteins of *Acidianus*, *Metallosphaera*, and *Sulfolobus* species. We also found an extra copy of SoxA and SoxB together in a distant region of the genome and a third copy of SoxB flanked by proteins with unknown function. Regarding SoxD, there was only one accession in NCBI at the time of writing; a quinol oxidase subunit (BAK54160) deduced from the complete genome sequence of *S. tokodaii* str. 7 [41], which is a very small putative protein (43 amino acids) with unknown function. *A. copahuensis* soxD homologous is located in the *sox* cluster and also codes for a small putative protein with 65% sequence identity to the SoxD of *S. tokodaii*. The SoxABCD complex functions as a terminal quinol oxidase (the heme *aa*_3_-CuB centre is the site of oxygen reduction) [42] and it was upregulated in *M. sedula* grown on sulfur compared to yeast extract or pyrite [43]. In *A. copahuensis* genome, as well as in the annotated genomes of other Sulfolobales, between *soxD* and *soxA* there is a gene *soxL2* coding for a 2Fe-2S Rieske protein, which is putatively involved in electron transport [10].

As mentioned above *A. copahuensis* has homologous proteins to the *cbsAB/soxNL* cluster encoding a cytochrome *b* (SoxN), a 2Fe-2S Rieske protein (SoxL) and an analog to the cytochromes *c*_1_ (CbsA). The gene *cbsB* codes for a putative hydrophobic protein that has not been characterized yet [39]. The cluster is an analog to the *bc*_1_ cytochrome of Bacteria and Eukarya [39] and it was found upregulated in *M. sedula* and *S. acidocaldarius* grown on sulfur or pyrite compared to yeast extract. Such evidence suggests that it has an important role in chemolithotrophic metabolism [43–45].

We also found homologous of the *doxBCE* gene cluster that codes for the terminal quinol oxidase DoxBCE from *A. ambivalens* [46]. The purified enzyme consists of two subunits, DoxB and DoxC. DoxB is homologous of the heme *a* and heme *a*_3_-CuB bearing subunit I of terminal oxidases, although it has a very low sequence identity with other oxygen reductases. DoxC has even less similarity to biochemically characterized terminal oxidases and its function is not clear [47]. DoxE is a putative hydrophobic small protein of unknown function. *A. copahuensis* has two other copies of the *doxB* gene in distant parts of the genome that present between 60 and 65% identity with the *doxB* copy of the cluster and with *doxB* of *A. ambivalens*. The genomes of *M. sedula* and *S. tokodaii* also present multiple copies of the *doxB* gene, which could be indicative of an important metabolic function. *A. copahuensis* could be using the DoxBCE complex in the oxidation of sulfur and sulfur compounds, as it has been shown in other Sulfolobales [20].

Although we did not find in *A. copahuensis* similar genes to the terminal oxidase of the *soxM* supercomplex, associated with heterotrophic growth [43], we found two copies of a gene coding for proteins similar to the blue copper protein sulfocyanin (SoxE), involved in electron transfer between the subunits of the *soxM* supercomplex [48, 49]. The precise role of sulfocyanin is still not clear. Its participation in iron metabolism in archaea has been hypothesized for long [38, 50], however it has been only shown in *Ferroplasma acidiphilum* [37]. Further experimental work will be required to determine whether the sulfocyanin or the rusticyanin-like mentioned in the previous section play essential roles as electron transport intermediates in iron oxidation pathways.

#### Carbon fixation

We have experimentally demonstrated that *A. copahuensis* is able to fix carbon and grow autotrophically on sulfur or tetrathionate [11]. The ability to grow using CO_2_ from the atmosphere is extremely relevant for biomining microorganisms in order to avoid the need to add organic compounds in bioleaching/biooxidation operations.

Chemolithoautotrophic archaea fix carbon using pathways different from those in autotrophic bacteria or eukaryotes [51]. Members of the order Sulfolobales are able to grow autotrophically using the hydroxypropionate–hydroxybutyrate cycle [52], a pathway that at the moment has been only found in this group of organisms. The enzymes involved in this pathway are tolerant to oxygen and therefore can be used by aerobic, anaerobic, and facultative anaerobic Sulfolobales species. Similarly to other carbon fixation pathways in archaea, CO_2_ is fixed through the acetyl-CoA synthesis (from two bicarbonate molecules). A total of 19 different genes have been proposed to be involved in the hydroxypropionate–hydroxybutyrate pathway (Table 3), where the key enzymes are the acetyl-CoA propionyl-CoA carboxylase (the CO_2_-fixing enzyme), methylmalonyl-CoA mutase, and 4-hydroxybutyryl-CoA dehydratase. All genes, except the *hbcS*1 gene (which is only found in *Metallosphaera* species) were found in *A. copahuensis* (Table 3). In addition, we found the presence of the 18 bp HhcR regulatory motif [53] at the promoters of 13 genes (and relatively close to the start codon in other two genes) as well as the corresponding gene coding for the HhcR transcription factor (Table 3). All together, these results indicate that this strain is also able to fix carbon through the hydroxypropionate–hydroxybutyrate pathway.

**Table 3:** Carbon fixation proteins from the hydroxypropionate–hydroxybutyrate carbon fixation pathway in *M. sedula* and the homologous found in *A. copahuensis* genome

	*Metallosphaera sedula*		*Acidianus copahuensis*					
Gene name	Uniprot ID	ORF (aa)	NCBI accession	ORF (aa)	E-value	Aa identical positions (%)	HhcR (position)	Protein Function
*HhcR*	A4YIR2	115	EZQ01893	115	1.00E^-46^	64		HhcR transcription factor
*accA*	A4YD22	510	EZQ11106	507	0	78	+ (−46)	Acetyl-CoA/propionyl-CoA carboxylase, alpha subunit
*accB*	A4YD23	167	EZQ11107	167	2.00E^-84^	64	-	Acetyl-CoA/propionyl-CoA carboxylase, beta subunit
*accC*	A4YGI1	524	EZQ11108	523	0	81	+ (−53)	Acetyl-CoA/propionyl-CoA carboxylase, gamma subunit
*mcr*	A4YEN2	357	EZQ11004	356	0	82	? (−274)	Malonyl-CoA/succinyl-CoA reductase
*msr*	A4YI81	314	EZQ04857	317	2.00E^-174^	75	+ (−20)	Malonate semialdehyde reductase
*hpcS*	A4YGR1	661	EZQ11066	653	0	78	-	3-Hydroxypropionyl-CoA synthetase
*acr*	A4YGN2	332	EZQ01730	333	0	76	+ (−19)	Acryloyl-CoA reductase
*mce*	A4YEG2	140	EZQ10835	141	4.00E^-71^	70	+ (−65)	Methylmalonyl-CoA epimerase
*mcmA*	A4YEG1	553	EZQ10834	550	0	83	? (−242)	Methylmalonyl-CoA mutase (catalytic subunit)
*mcmB*	A4YIE3	155	EZQ04694	138	8.00E^-75^	78	+ (−39)	Methylmalonyl-CoA mutase (coenzyme B12-binding subunit)
*ssr*	A4YGN0	360	EZQ01731	360	0	77	+ (−41)	Succinate semialdehyde reductase
*hbcS1*	A4YDR9	549	-	-	-	-	-	4-Hydroxybutyryl-CoA synthetase
*hbcS2*	A4YDT1	564	EZQ11368	559	0	58	-	4-Hydroxybutyryl-CoA synthetase
*hbcS3*	A4YGM8	472	EZQ01733	471	0	85	+ (−37)	4-Hydroxybutyryl-CoA synthetase
*hbcD*	A4YGC7	507	EZQ02033	506	0	75	+ (−30)	4-Hydroxybutyryl-CoA dehydratase
*ack*	A4YEH9	395	EZQ11076	396	0	71	+ (−40)	Acetoacetyl-CoA-ketothiolase
*hbd2*	A4YGM9	391	EZQ01732	388	0	72	+ (−46)	Probable 3-hydroxybutyryl-CoA dehydrogenase
*hpcD-hbd*	A4YDS4	651	EZQ11226	661	0	49	+ (−91)	Bifunctional crotonyl-CoA hydratase/3-hydroxybutyryl-CoA dehydrogenase
	A4YI89	259	EZQ04864	257	2.00E^-133^	71	+ (−15)	3-hydroxypropionyl-coenzyme A dehydratase

The proteins are identified by their NCBI accession number (*A. copahuensis*) or Uniprot identification number (*M. sedula*). ORF (aa) indicates length of the open reading frame in amino acids, E-values and amino acid identities were obtained by BLASTP analysis. The presence of the HhcR regulatory motifs in *A. copahuensis* at the promoter of each gene was determined following the procedures described by Leyn et al. 2015 [53]. Presence is indicated with (+), absence by (−) and cases where it was found more distant than average, with (?), and positions upstream the start codon are indicated in parenthesis. Aa: amino acids

#### Metal and metalloids resistance

Biomining involves the accumulation of metals, especially heavy metals, most of which are toxic for life forms. Hence, several microorganisms have developed strategies, such as mobilization, chelation and transformation, to deal with these compounds. Although these mechanisms as well as the genes involved are not fully known [54], it has been suggested that they could act by either having a direct effect on metal compounds or by triggering stress responses that protect cells from toxicity [55]. *A. copahuensis* as well as most other Sulfolobales have a three genes cluster containing the *copA, copM* and *copT* genes that are involved especially in copper but also in zinc and cadmium transport (Additional file 3: Table S2) [56–58]. Resistance to copper is of special interest as thermoacidophilic archaea such as *A. copahuensis* [7, 59], can dissolve different copper sulfides including chalcopyrite, the world’s largest copper reservoir [5]. All sequenced Sulfolobales species also use a mechanism to sequestrate metal cations on inorganic polyphosphates (polyP) forming a complex that is then excreted [60]. Although all genes involved in this mechanism are not fully elucidated, we detected in *A. copahuensis* (and in all other Sulfolobales genomes) homologous for all genes that had been previously associated to this process.

Arsenic is also often present in biomining operations since many ores have a great content of arsenosulfides (mainly arsenopyrite), which can be solubilized simultaneously with the metal sulfide of interest. Arsenic is a toxic compound for most organisms on earth. However, certain groups of prokaryotes are able to survive in presence of arsenic by using a detoxification mechanism based on the ars operon (ArsABCDR enzymes). Some other microorganisms are also able to use arsenic in bioenergetic processes such as anaerobic respiration of arsenate (using in most cases the ArrA enzyme but also other yet unknown enzyme in some Crenarchaeota species) and chemolithotrophic growth on arsenite (using the AioAB enzymes) [61]. In *A. copahuensis* genome we found genes coding for the arsenite oxidase large molydobpterin subunit (*aioA*) and small Rieske-like subunit (*aioB*). Both genes were located next to each other similarly to all organisms with functional arsenite oxidases. Phylogenetic analysis of the *aioA* confirmed the identity of the enzyme and showed that all *aioA* from Sulfolobales clustered together and clearly separated from other orders in phylum Crenarchaeota, from the phylum Euryarchaeota and from all Bacteria (Additional file 7: Figure S4). Similarly to what was observed for the SOR enzyme, the phylogeny of the *aioA* resembles the evolutionary history of the main prokaryotic lineages and has been thus proposed as an ancient enzyme emerged in the most primitive life forms [62, 63]. It is worth noting that *aioA* genes are only present in 6 other genomes from the 14 available for Sulfolobales species.

From all other known arsenic related enzymes, we only found one gene with high similarity to the ArsB enzyme (Additional file 3: Table S2), a membrane protein used to transport arsenite out of the cell and some ArsR-like ORFs. Although the ArsB protein is commonly found within the *ars* operon, none of the other enzymes (i.e., ArsA, ArsC, ArsD) were found in *A. copahuensis* genome. This observation was also true to all other Crenarchaeota genomes available in NCBI database, in which only *arsB* was found among all *ars* operon enzymes. These results suggest that *A. copahuensis* could be used in bioremediation of arsenic contamination, as arsenite is more toxic and more mobile than arsenate. Moreover, it could be possible that *A. copahuensis* is able to gain energy from arsenite, thus enabling chemolithotrophic growth on arsenite, through the oxidation of arsenite by *aioAB* genes. They also suggest that in *A. copahuensis*, similarly to other Crenarchaeota, arsenite pumps would work as the sole arsenic detoxification mechanism, although we cannot rule out the possibility of a synergic interaction between ArsB and AioAB (both membrane proteins) to optimize bioenergetics processes involving the oxidation of arsenite or other yet unknown arsenic detoxifying mechanisms.

## Conclusion

In this work we identified the genetic pathways that are most likely responsible of the biomining metabolic features that we had previously characterized experimentally in *Acidianus copahuensis*, such as sulfur and iron oxidation, carbon fixation and heavy metals and metalloids resistance. While some of these features are likely common to all Sulfolobales, others are only found in certain lineages (e.g., iron oxidation). Among thermoacidophilic archaea, *Acidianus copahuensis* seems to be a remarkable candidate for biomining activities as it contains nearly all biomining related features that were previously identified in this group and many other yet uncharacterized genes that are uniquely found in this species. In addition, *A. copahuensis* seems to be a good candidate for the study of relevant, yet poorly characterized, metabolic pathways such as sulfur compounds oxidation through the DsrE, TusA and Hdr-like complex as well as the role in iron oxidation of the *fox* cluster and the rusticyanin-like protein in thermoacidophilic archaea. The in-depth genome characterization presented in this work will certainly lead to a better comprehension of the biology of *Acidianus copahuensis* and Sulfolobales in general, and together with experimental data, to the discovery of novel metabolic functions with biotechnological potential for biomining activities.

## Methods

### Cultivation of a. Copahuensis and DNA extraction

*A. copahuensis* ALE1 strain was cultured in 100 mL flasks in MAC medium [64] at initial pH of 3 supplemented with sulfur powder (10 g/L) and yeast extract (1 g/L) at 65 °C in agitation (150 rpm). After 10 days cells were harvested by centrifugation from 10 mL of culture. The pellet was resuspended by vortexing with 20% *w*/*V* sucrose solution in TE buffer (10 mM Tris HCl pH 8.0, 1.0 mM EDTA) and then treated with 250 μL of 5 μg/mL lysozyme in TE buffer solution at 37 °C for 1 h. To improve cell lysis 100 μL of solution of protease K 5 mg/mL and SDS 10% in TE buffer were added and incubated 1 h at 37 °C. In order to separate aqueous and organic phases 70 μL sodium acetate 3.0 M (pH 3.4), 100 μL of chloroform and 200 μL phenol–Tris were added and centrifuged 15 min at 13,500 rpm. Aqueous phase was extracted using 100 μL of chloroform and centrifuged 15 min at 13,500 rpm for decanting cellular rests. Cold isopropyl alcohol was used for DNA precipitation in 1 h incubation at −20 °C and 20 min centrifugation at 13,500 rpm using a refrigerated (4 °C) centrifuge. DNA pellet was washed with 70% cold ethanol and air dried. DNA was resuspended in 50 μL of Tris-HCl 10 mM pH 8 buffer and incubated at 60 °C for 1 h. DNA concentration was measured in a NanoDrop spectrophotometer and its integrity was evaluated in a 0.8% agarose gel electrophoresis stained with ethidium bromide.

### Bioinformatic analyses

The genome of *Acidianus copahuensis* was recently published by our group [65] and is publicly available in NCBI under the accession number JFZT00000000, with the corresponding annotation obtained by the NCBI Prokaryotic Genome Annotation Pipeline. We also uploaded the *A. copahuensis* genome together with all 13 others available from the order Sulfolobales to the RAST server [66] to get an alternative annotation. Sequences from the metabolisms of interest were identified using a combination of annotations from RAST/NCBI and BLASTP analysis against known representative sequences of these metabolisms. For the latter, only hits with very low E-values (< 1e^−20^) were considered and candidates were validated against the full NCBI Database by BLASTP. Additionally, the adjacent genes to the genes of interest were also analyzed in comparison to closely related genomes to validate the loci genomic organization. All Blast searches against the NCBI nr database were performed in mid 2015.

The digital DDH analysis to compare the 14 available Sulfolobales genomes was performed using the GGDC 2 method [67]. The proteins predicted by the RAST server in all these genomes were compared to each other by BLASTP (all vs. all analysis) and only hits with E-value lower than 1E-20 and at least 65% of the length of both proteins aligned were conserved. Based on these results, for each protein-coding gene in *A. copahuensis,* it was determined in which other genomes the proteins were found and a presence/absence table was built. These results were visualized with a heatmap analysis (heatmap.2 function) in R using the Euclidean distance for clustering. Network analysis was performed using the EGN software [68] using the same similarity thresholds than for filtering BLASTP results and visualization and plot was made in Cytoscape.

For the cases where genome organization of certain gene complexes were compared between different species (i.e., *fox* and *dsr* genes), we performed a de novo ORF prediction using FragGeneScan [69] to get the genome positions, gene orientations and gene sizes. All predicted peptides were then compared by BLASTP to a reference database with the genes of interest and then hits validated by reciprocal best hits between pairs of genomes. The percentages of similarity between proteins were obtained from BLASTP analysis. For phylogenetic analyses we aligned sequences using T-COFFEE [70] with default parameters and constructed Maximum likelihood (ML) trees in MEGA6 software [71]. Best ML substitution model was estimated prior to phylogenetic analysis also in MEGA6. Bootstrap support for the obtained trees was estimated using 1000 repetitions.

## Additional files

Additional file 1: Figure S1. Pairwise comparison indicating the number of proteins shared between all Sulfolobales genomes. Comparison was performed by BLASTP and only hits with E-value lower that 1E-20 and more than 65% of both proteins aligned were considered as a match. Values correspond to percentage of total proteins in the query genome. (PDF 36 kb)

Additional file 2: Table S1. Complete information on the genes predicted in the *Acidianus copahuensis* genome based on RAST server processing results. The table contains all genomic positions from all predicted genes, with their corresponding sequences and annotations, the Sulfolobales species where they were also found, and the color references used in Fig. 1b. (XLSX 1646 kb)

Additional file 3: Table S2. List of proteins found in *A. copahuensis* corresponding to relevant metabolisms for biomining activities that are mentioned throughout this work. (XLSX 12 kb)

Additional file 4: Figure S2. Schematic representation of the putative proteins found in *A. copahuensis* genome related to sulfur oxidation. The way sulfur and tetrathionate enter the cytoplasm is still unknown. SOR: sulfur oxygenase reductase. SQR: sulfide:quinone oxidoreductase. TQO: thiosulfate quinone oxidoreductase. Sulfite oxidase: sulfite oxidase-like protein, its subcellular location and electron transference mechanisms in *A. copahuensis* are yet unknown (indicated by “?”). TTH: Tetrathionate hydrolase, the exact location of the enzyme is still not clear. Q and QH_2_: oxidized and reduced quinones, respectively. Hdr: heterodisulfide reductase, the exact location of the enzyme is still not clear. The complete mechanism of sulfur compounds oxidation mediated by Hdr, TusA and DsrE proteins is not clear yet, other possible reactions have been omitted for clarity (for more detail see text and references mentioned there). (JPEG 97 kb)

Additional file 5: Figure S3. Phylogenetic analysis of rusticyanin-like and sulfocyanin-like proteins from *Acidianus copahuensis*. Phylogenetic trees were obtained by the Neighbor Joining method. Bootstrap supports for nodes were obtained using 1000 repetitions and are expressed as the proportion of times (in decimals) that each node was supported. Sequences are compared to well characterized sulfocyanin and rusticyanin proteins to validate the classifications of those in *A. copahuensis*. NCBI accession numbers of each protein in the tree are indicated at the beginning of each branch name. (PDF 2 kb)

Additional file 6: Table S3. Comparison between the Fox proteins described in *S. metallicus* [9] and the homologous found in *A. copahuensis* genome. The proteins are identified by their NCBI accession number. ORF (aa): indicates length of the open reading frame in amino acids. (XLSX 9 kb)

Additional file 7: Figure S4. Phylogeny of the large subunit of arsenite oxidases (AioA). Phylogenetic trees were obtained using the Maximum Likelihood method with all known AioA proteins in prokaryotes. The different lineages were collapsed to facilitate tree interpretation and to show the positioning of Sulfolobales lineages among all taxonomic groups with AioA. Sulfolobales proteins were re-computed separately and are represented in the squared figure in the lower-left panel of the figure. Bootstrap supports for nodes were obtained using 1000 repetitions and are expressed as the proportion of times (in decimals) that each node was supported. (PDF 44 kb)

## Abbreviations

BLAST: Basic local alignment search tool
DDH: DNA-DNA hybridization
GGDC: Genome to genome distance calculator
Hdr: Heterodisulfide reductase
KEGG: Kyoto encyclopedia of genes and genomes
NCBI: National center for biotechnology information
ORF: Open reading frame
RAST: Rapid annotation using subsystem technology
SOR: Sulfur oxygenase reductase
SQR: Sulfide:quinone oxidoreductase
TQO: Thiosulfate:quinone oxidoreductase
TTH: Tetrathionate hydrolase
