# Supplementary material for: Genome analysis of the thermoacidophilic archaeon Acidianus copahuensis focusing on the metabolisms associated to biomining activities
Source: BMC Genomics. 2017 Jun 6;18:445. doi: 10.1186/s12864-017-3828-x (PMC5461723; doi:10.1186/s12864-017-3828-x)
Supplement: Supplementary file 1 — Pairwise comparison indicating the number of proteins shared between all Sulfolobales genomes. Comparison was performed by BLASTP and only hits with E-value lower that 1E-20 and more than 65% of both proteins aligned were considered as a match. Values correspond to percentage of total proteins in the query genome. (PDF 36 kb) [file 12864_2017_3828_MOESM1_ESM.pdf]

Target Genome

Query Genome	*Acidianus copahuensis*	*Acidianus hospitalis W1*	*Metallosphaera cuprina*	*Metallosphaera hakonensis*	*Metallosphaera sedula*	*Metallosphaera yellowstonensis*	*Sulfolobales archaeon AZ1*	*Sulfolobales archaeon Acd1*	*Sulfolobus acidocaldarius*	*Sulfolobus islandicus*	*Sulfolobus metallicus*	*Sulfolobus solfataricus*	*Sulfolobus sp.JCM16833*	*Sulfolobus tokodaii*
Acidianus copahuensis		66	64	64	68	67	63	50	61	65	58	67	61	68
Acidianus hospitalis W1	66		59	61	64	63	63	52	60	65	58	66	62	72
Metallosphaera cuprina	76	72		81	85	81	72	59	69	74	65	74	68	75
Metallosphaera hakonensis	58	56	61		67	65	54	45	54	58	51	59	54	60
Metallosphaera sedula	69	65	73	75		76	64	53	63	68	58	69	61	70
Metallosphaera yellowstonensis	50	49	50	55	57		46	40	47	55	44	54	46	54
Sulfolobales archaeon AZ1	76	76	73	70	75	74		60	70	73	66	73	69	76
Sulfolobales archaeon Acd1	70	72	68	68	71	73	69		70	72	65	73	67	76
Sulfolobus acidocaldarius	65	64	62	63	66	66	62	54		72	57	73	61	74
Sulfolobus islandicus	59	58	55	57	60	62	54	46	60		52	78	54	67
Sulfolobus metallicus	53	53	49	50	52	52	50	42	48	52		53	62	54
Sulfolobus solfataricus	55	55	50	55	57	60	50	43	56	75	49		52	64
Sulfolobus sp.JCM16833	54	54	50	52	53	53	51	42	50	55	61	55		57
Sulfolobus tokodaii	59	62	54	58	60	60	54	48	60	65	52	66	56	
