# Supplementary figures and images for: Genome analysis of the thermoacidophilic archaeon Acidianus copahuensis focusing on the metabolisms associated to biomining activities
Source: BMC Genomics. 2017 Jun 6;18:445. doi: 10.1186/s12864-017-3828-x (PMC5461723; doi:10.1186/s12864-017-3828-x)

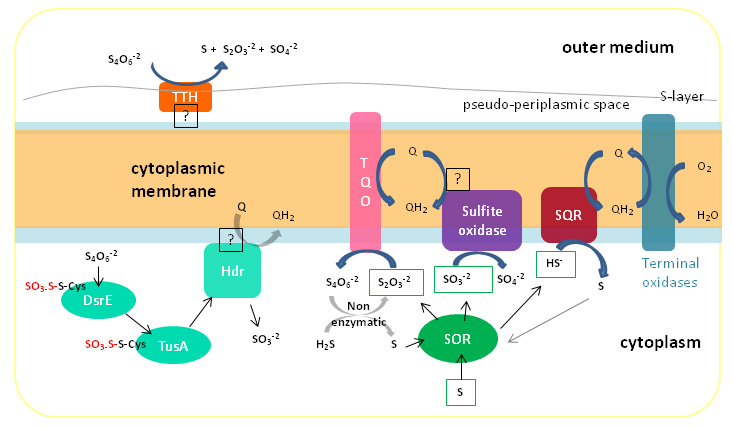

Supplement: Supplementary file 4 — Schematic representation of the putative proteins found in A. copahuensis genome related to sulfur oxidation. The way sulfur and tetrathionate enter the cytoplasm is still unknown. SOR: sulfur oxygenase reductase. SQR: sulfide:quinone oxidoreductase. TQO: thiosulfate quinone oxidoreductase. Sulfite oxidase: sulfite oxidase-like protein, its subcellular location and electron transference mechanisms in A. copahuensis are yet unknown (indicated by “?”). TTH: Tetrathionate hydrolase, the exact location of the enzyme is still not clear. Q and QH2: oxidized and reduced quinones, respectively. Hdr: heterodisulfide reductase, the exact location of the enzyme is still not clear. The complete mechanism of sulfur compounds oxidation mediated by Hdr, TusA and DsrE proteins is not clear yet, other possible reactions have been omitted for clarity (for more detail see text and references mentioned there). (JPEG 97 kb) [file 12864_2017_3828_MOESM4_ESM.jpg]

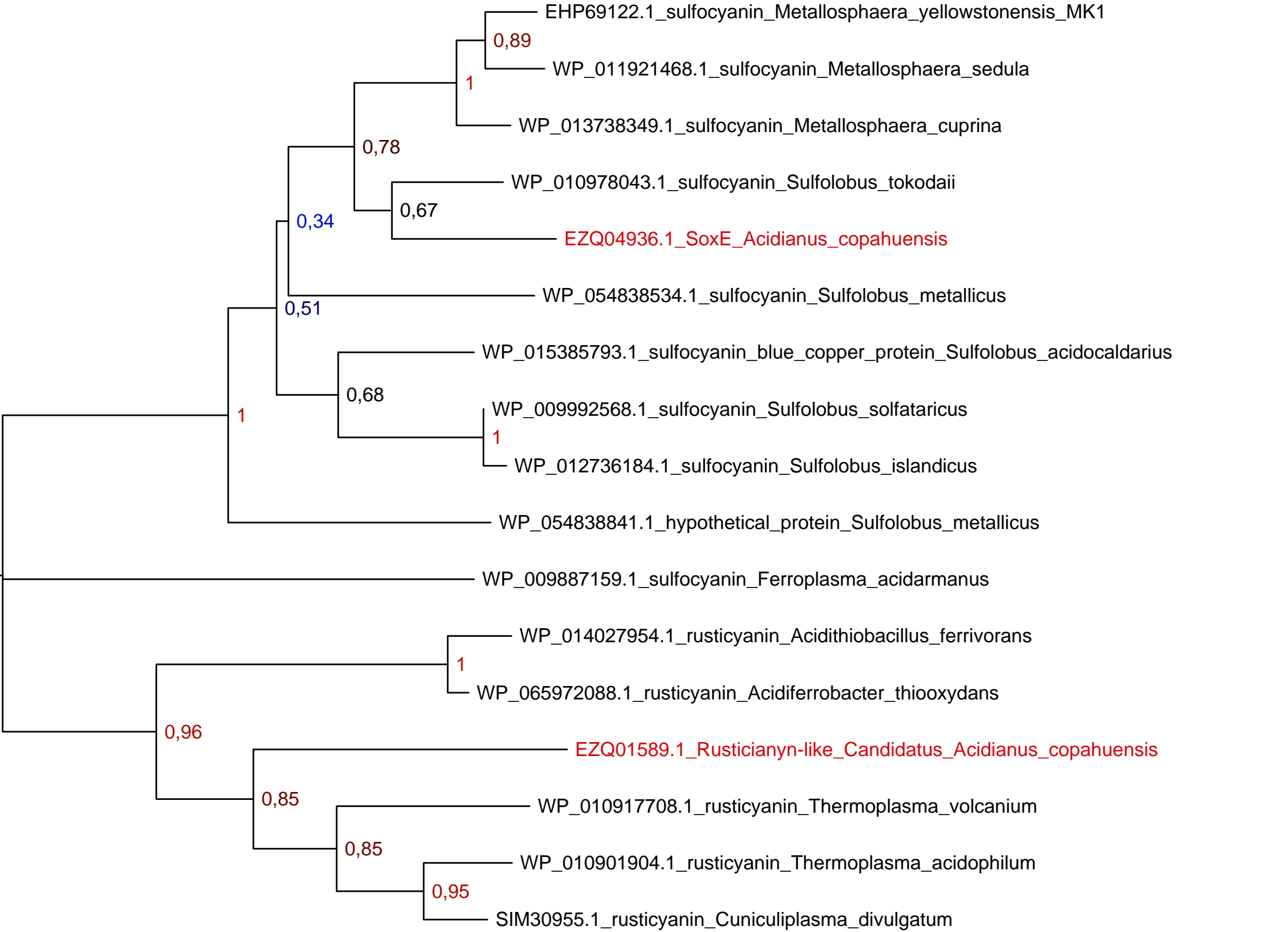

0.08

Supplement: Supplementary file 5 — Phylogenetic analysis of rusticyanin-like and sulfocyanin-like proteins from Acidianus copahuensis. Phylogenetic trees were obtained by the Neighbor Joining method. Bootstrap supports for nodes were obtained using 1000 repetitions and are expressed as the proportion of times (in decimals) that each node was supported. Sequences are compared to well characterized sulfocyanin and rusticyanin proteins to validate the classifications of those in A. copahuensis. NCBI accession numbers of each protein in the tree are indicated at the beginning of each branch name. (PDF 2 kb) [file 12864_2017_3828_MOESM5_ESM.pdf]

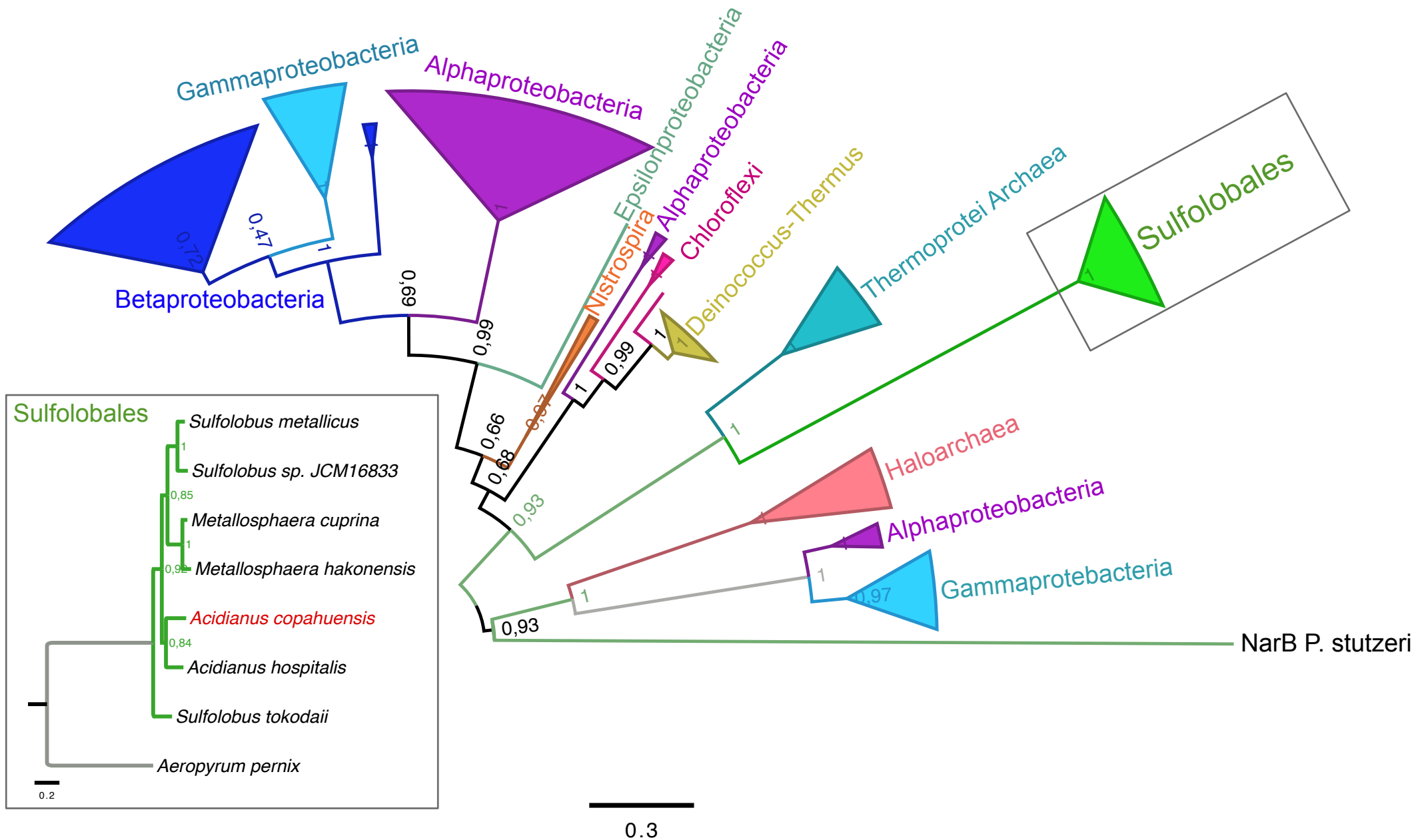

Supplement: Supplementary file 7 — Phylogeny of the large subunit of arsenite oxidases (AioA). Phylogenetic trees were obtained using the Maximum Likelihood method with all known AioA proteins in prokaryotes. The different lineages were collapsed to facilitate tree interpretation and to show the positioning of Sulfolobales lineages among all taxonomic groups with AioA. Sulfolobales proteins were re-computed separately and are represented in the squared figure in the lower-left panel of the figure. Bootstrap supports for nodes were obtained using 1000 repetitions and are expressed as the proportion of times (in decimals) that each node was supported. (PDF 44 kb) [file 12864_2017_3828_MOESM7_ESM.pdf]
